# Recurrent Multiple Cervical Esophageal Webs: An Unusual Presentation of Celiac Disease

**DOI:** 10.4021/gr2009.12.1325

**Published:** 2009-11-20

**Authors:** Usha Dutta, Abdul Khaliq, Mohd Talha Noor, Rakesh Kochhar, Kartar Singh

**Affiliations:** a Departments of Gastroenterology, Postgraduate Institute of Medical Education and Research (PGIMER), Chandigarh, India

**Keywords:** Esophageal web, Celiac disease, Iron deficiency anemia

## Abstract

Although the association of celiac disease with esophageal web has been reported earlier, in this case patient presented with persistent dysphagia. Upper gastrointestinal endoscopy revealed multiple esophageal webs which were recurring despite endoscopic dilatation. Diagnosis and treatment of underlying celiac disease led to long term improvement.

## Introduction

Cervical web is a common benign cause of dysphagia. Endoscopic fracture/dilatation of the web results in resolution of the symptoms. Recurrence of cervical web is rare. Cervical web is usually associated with iron deficiency anemia. Celiac disease is a common cause of refractory iron deficiency anemia in North India. We describe a 35 year old man with recurrent cervical webs with severe iron deficiency anemia, secondary to celiac disease who responded dramatically to gluten free diet.

## Case Report

A 35 years old man presented with symptoms of dysphagia of 5 years duration to solids which was insidious in onset and persistent. His appetite was normal but had weight loss secondary to decreased intake. There was no history suggestive of reflux disease, drug ingestion or corrosive intake. His bowel habits were normal. He underwent 4 sessions of esophageal dilations by surgeons under general anaesthesia, but the webs recurred and he was referred to gastroenterology services.

**Figure 1:**
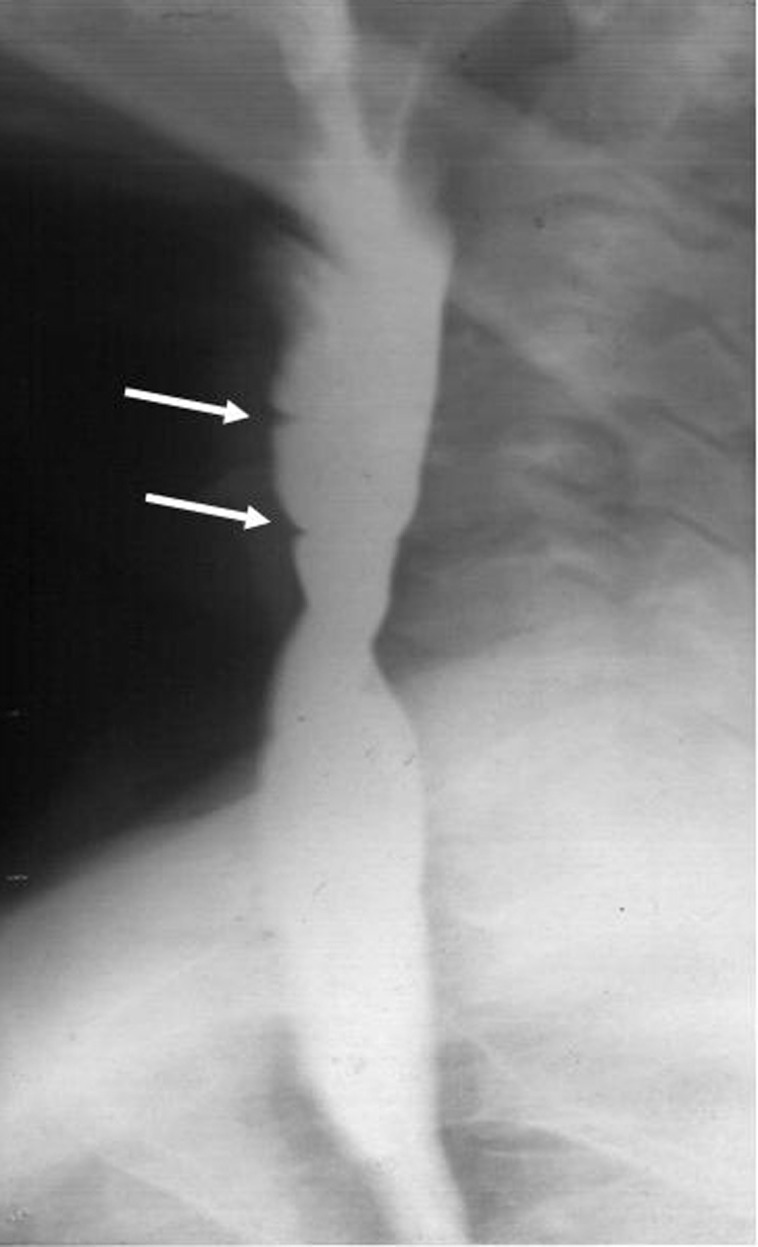
Barium swallow examination showing evidence of multiple webs in the upper esophagus (arrows).

**Figure 2:**
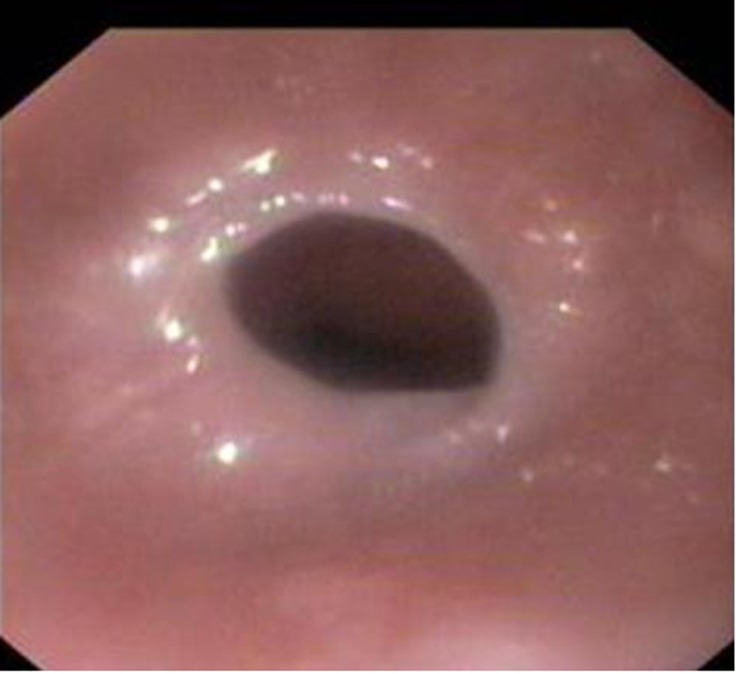
Upper gastrointestinal endoscopic image showing a web in the upper esophagus.

On evaluation he was thin built and pale. General physical examination was unremarkable except for pallor. Barium swallow showed multiple esophageal webs in the post cricoid region (Fig. 1). He had microcytic and hypochromic anemia with a hemoglobin of 8.2g%. Stool for occult blood was negative and iron studies showed severe iron deficiency state with serum iron of 13 micrograms/dl and percent transferrin saturation of 2.7%. Upper gastro-intestinal endoscopy showed multiple cervical webs and scalloping and grooving of the proximal duodenum (Fig. 2). A possibility of celiac was considered and duodenal biopsy was done. Serum tissue transglutaminase (tTG) was elevated at 28 U/ml (normal < 10 U/ml) and the duodenal biopsy showed villous atrophy with increase in the intra-epithelial lymphocytes consistent with the diagnosis of celiac disease. He was advised gluten free diet and iron supplementation. On this treatment his dysphagia was resolved, and there was no recurrence of webs. He gained 6 kilograms of weight, and his anemia and serum iron profile also started showing improvement (hemoglobin 12g%, serum iron 53 mg/dl, and percent transferring saturation 9.8%) within six weeks of treatment. He developed a pruritic papular skin rash over extensor aspects of limbs suggestive of dermatitis herpertiformis which responded to Dapsone therapy. He has been doing well since then and is asymptomatic on 7 years of follow-up.

## Discussion

Most of the esophageal webs are asymptomatic and only a few cause dysphagia [1]. Esophageal webs are usually single, in the upper one third of the esophagus and respond completely to single time dilatation [2]. Recurrent cervical webs are uncommon and usually associated with iron deficiency. Other conditions associated with esophageal webs are autoimmune disorders of thyroid, pernicious anemia, graft versus host disease, rheumatoid arthritis, psoariasis and blistering diseases of the skin [3, 4]. Association of celiac disease with esophageal web has been reported in literature [5-7]. In this case there were multiple esophageal webs which were recurrent and refractory to multiple sessions of dilatation. Detection and treatment of underlying celiac disease led to long term resolution of esophageal webs and improvement in anemia.

In India, the commonest cause of iron deficiency is nutritional inadequacy and worm infestation. However, in North India, celiac disease is very important cause of refractory iron deficiency anemia [8]. All patients with refractory iron deficiency anemia should be evaluated for celiac disease especially in high incidence populations [9]. A high index of suspicion for diagnosis of celiac disease is necessary in patients with iron deficiency anemia or esophageal web. Institution of gluten free diet in these patients provides gratifying results.
